# Matched Short-Term Depression and Recovery Encodes Interspike Interval at a Central Synapse

**DOI:** 10.1038/s41598-018-31996-0

**Published:** 2018-09-11

**Authors:** Armando E. Castillo, Sergio Rossoni, Jeremy E. Niven

**Affiliations:** 1 School of Life Sciences and Centre for Computational Neuroscience and Robotics, University of Sussex, Falmer, Brighton, BN1 9QG UK; 2 Centro de Neurociencias, Instituto de Investigaciones Científicas y Servicios de Alta Tecnología, Ciudad de Saber, Republic of Panama; 3 Present Address: Department of Physiology, Development and Neuroscience, University of Cambridge, Cambridge, CB2 3EJ UK

## Abstract

Reversible decreases in synaptic strength, known as short-term depression (STD), are widespread in neural circuits. Various computational roles have been attributed to STD but these tend to focus upon the initial depression rather than the subsequent recovery. We studied the role of STD and recovery at an excitatory synapse between the fast extensor tibiae (FETi) and flexor tibiae (flexor) motor neurons in the desert locust (*Schistocerca gregaria*) by making paired intracellular recordings *in vivo*. Over behaviorally relevant pre-synaptic spike frequencies, we found that this synapse undergoes matched frequency-dependent STD and recovery; higher frequency spikes that evoke stronger, faster STD also produce stronger, faster recovery. The precise matching of depression and recovery time constants at this synapse ensures that flexor excitatory post-synaptic potential (EPSP) amplitude encodes the presynaptic FETi interspike interval (ISI). Computational modelling shows that this precise matching enables the FETi-flexor synapse to linearly encode the ISI in the EPSP amplitude, a coding strategy that may be widespread in neural circuits.

## Introduction

The short-term depression (STD) of synaptic strength has been proposed to perform a variety of computational roles in neural circuits, including low pass filtering of synaptic inputs^1–3^, and reducing sensitivity to presynaptic activity^1^. Despite the importance of STD in neural circuits, the computational role of STD remains debated^4^. Many and varied computational roles that have been attributed to STD, and it is likely that it has different functions depending on the synapse at which it is found^1–4^. However, many of computational roles assigned to STD are based on its effects upon the steady-state excitatory post-synaptic potential (EPSP) amplitude, rather than exploring the rapid changes in synaptic strength that STD can produce.

Even when the impact of STD on computation at a specific synapse can be characterized, this is often based upon *in vitro* electrophysiological recordings making it difficult to relate to conditions *in vivo*. Moreover, the majority of studies have quantified the initial reduction of synaptic strength in detail, whilst the subsequent recovery and how it affects the processing and transmission of information at synapses is often considered only in terms of a single time constant. Yet studies of some synapses have shown that recovery dynamics can be as varied as the depression itself and are likely to have substantial effects upon synaptic dynamics^5–9^.

**Figure 1:**
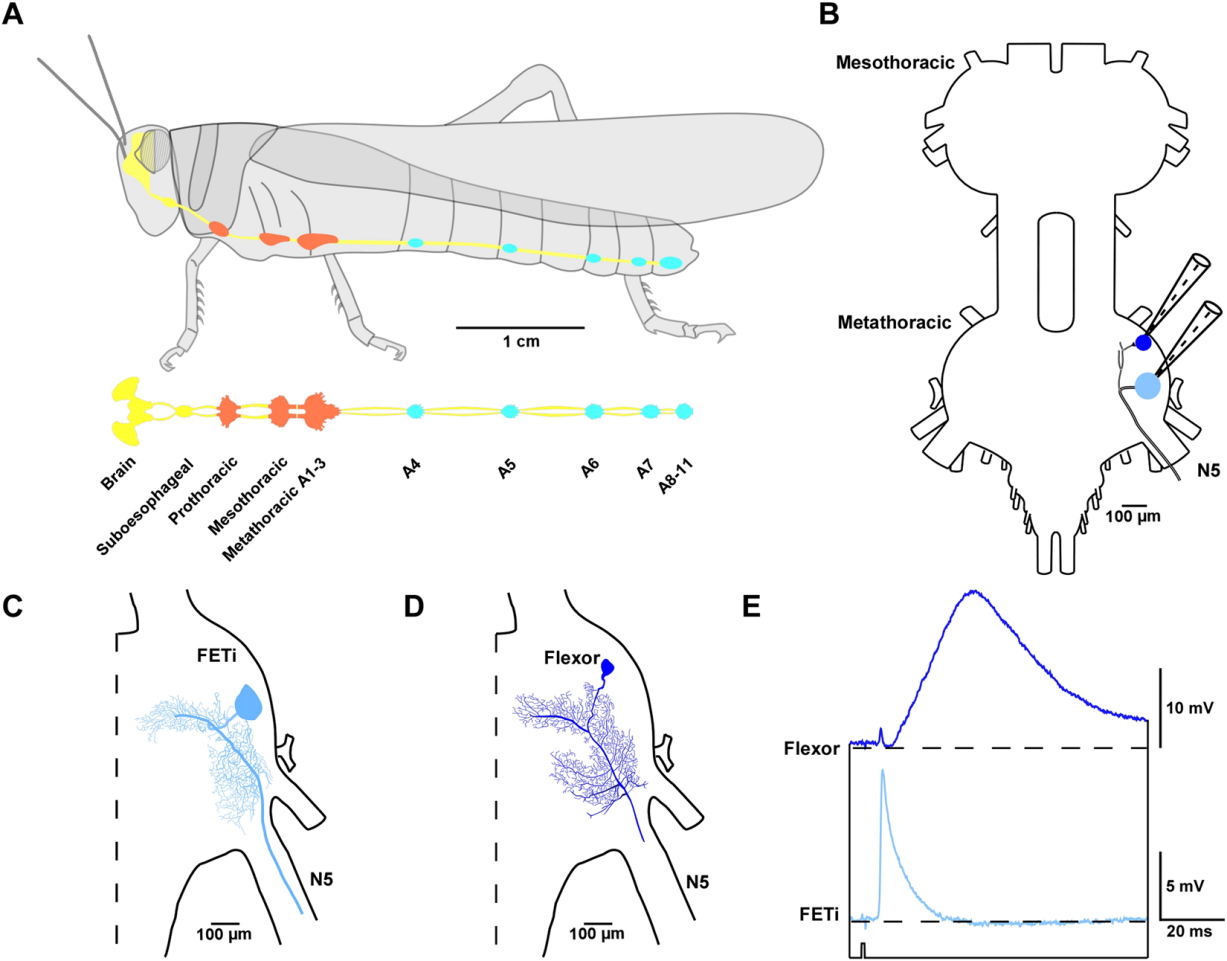
A synaptic connection exists between the fast extensor tibia (FETi) and flexor motor neurons in the desert locust. (**A**) A schematic diagram of the lateral view of the desert locust and central nervous system (CNS) (above). A dorsal view of the CNS is also shown (below). The brain and connectives are shown in yellow, thoracic ganglia in red and abdominal ganglia in blue. (**B**) An outline of the meso- and metathoracic ganglia showing the FETi and flexor recording sites in the metathoracic ganglion. (**C**) The central morphology of FETi showing the soma, dendrites, primary neurite and the axon exiting the ganglion through nerve 5 (N5). (**D**) The central morphology of a fast flexor. (**E**) An antidromic spike in FETi (pale blue) evokes an EPSP in a fast flexor (dark blue). The black dashed line indicates the resting potential of each neuron. The lower black line indicates the timing of the electrical stimulus that triggers the antidromic spike in FETi.

To determine the computational role of both STD and recovery *in vivo*, we recorded intracellularly pre- and post-synaptically at a central synapse in the desert locust, *Schistocerca gregaria* (Fig. 1A,B)^6,10,11^. We recorded from a single presynaptic neuron, the fast extensor tibia motor neuron (FETi) (Figs 1C, S1A–D), and one of nine flexor tibiae motor neurons (flexors) in the metathoracic ganglion (Fig. 1D)^12^. In this ganglion, FETi forms a monosynaptic connection with the flexors, which can each be characterized as slow, intermediate or fast depending upon their electrical activity and the movements of the tibia that they evoke (Fig. 1E)^13^. As their names imply, FETi and the flexors innervate the antagonistic extensor tibiae and flexor tibiae muscles that control extension and flexion of the hind tibia, respectively.

Spikes generated in the FETi primary neurite^14^ spread to branches in the neuropile of the metathoracic ganglion (Figs 1C; S1A,B) where central output synapses are located, eliciting short-latency EPSPs in the flexors (Fig. S1A,B)^6,10^. However, EPSPs in the flexors can also be evoked by stimulating the peripheral terminals of FETi at the extensor tibiae muscle, eliciting a spike that travels antidromically along the axon (Figs 1E, S1C,D).

We show that matched frequency-dependent STD and recovery at the FETi–flexor synapse ensures that EPSP amplitude linearly encodes the presynaptic FETi inter-spike interval (ISI). Thus, flexor EPSP amplitude responds rapidly to shifts in FETi activity, permitting these motor neurons to contribute to motor patterns underpinning a wide range of behaviours.

## Results

### Frequency-Dependent Short-Term Depression and Recovery

Short-term depression at the FETi-flexor synapse can be studied over a range of behaviorally relevant frequencies by making use of the precise control of spike timing afforded by antidromic spikes^6,10,11^ (see Methods). We restricted our analyses to the connection between FETi and the fast flexors to minimize variation amongst our recordings. Single FETi spikes evoked 12–24 mV EPSPs (18.54 ± 0.92 mV; N = 16) in fast flexors, similar to those reported previously for both orthodromic and antidromic spikes at this synapse (Figs 1E, S1A–D).

**Figure 2:**
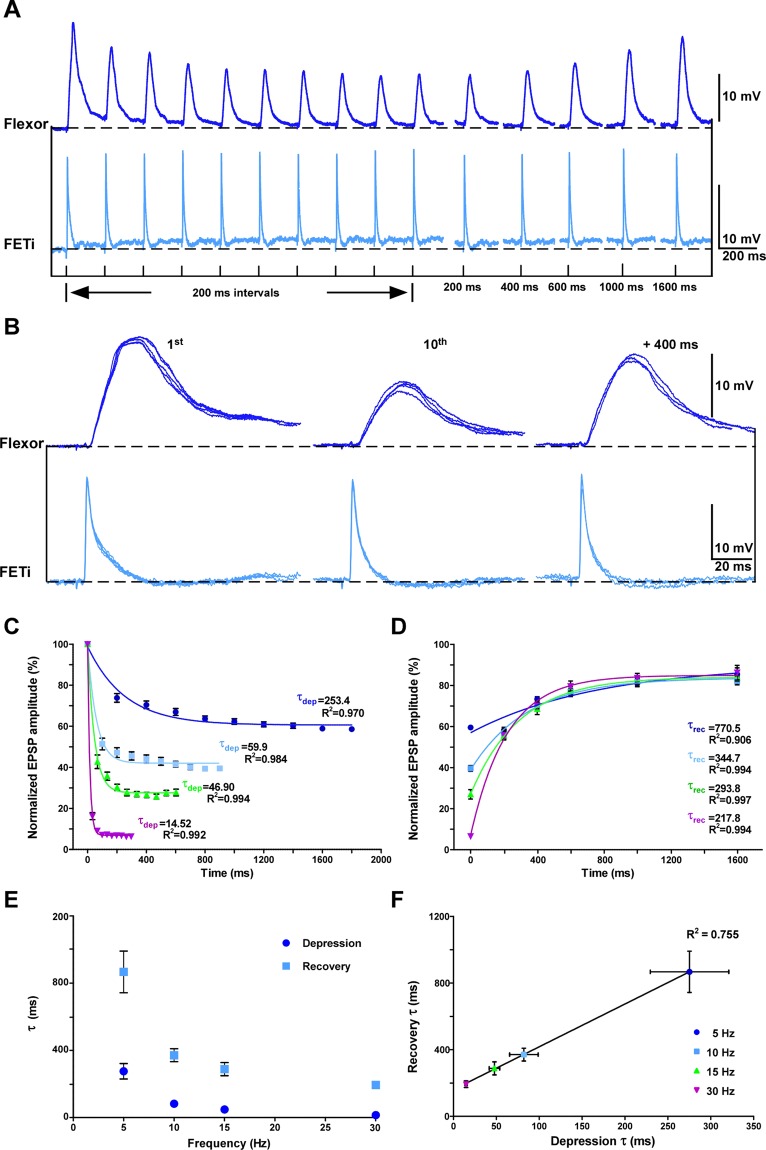
Matched frequency dependent short-term depression (STD) and recovery at the FETi-flexor synapse. (**A**) A paired intracellular recording of FETi (pale blue) and a flexor (dark blue). A train of 10 antidromic FETi spikes at 5 Hz cause STD in the flexor EPSP amplitude. The recovery is assessed with single antidromic spikes at intervals between 200 and 1600 ms. The stimuli evoking the antidromic spikes are shown below. (**B**) Overlays of 5 FETi spikes and the corresponding flexor EPSPs at different points during STD and recovery show the low inter trial variability of the EPSP amplitude. (**C**) The mean normalized amplitudes of flexor EPSPs evoked by trains of 10 antidromic FETi spikes at between 5 and 30 Hz. Data for each frequency are fitted with a single exponential. Data were obtained with 10 repeats of each stimulus in 16 animals. Error bars show the standard error of the mean (SEM). (**D**) The normalized mean amplitudes of flexor EPSPs evoked by single FETi spikes to assess recovery from 200 to 1600 ms. The recovery after trains of 10 antidromic FETi spikes at between 5 and 30 Hz is shown. Data for each frequency are fitted with a single exponential. Data were obtained with 2 repeats of each stimulus in 13 animals. (**E**) STD and recovery time constants as a function of the stimulation frequency. The time constants of the exponentials fitted to the STD and recovery are frequency dependent, decreasing as the spike frequency increases. Error bars show the SEM. (**F**) Recovery time constant *versus* depression time constant. The time constants of the exponentials fitted to the STD and recovery are matched to one another producing a linear relationship (Slope = 2.77, Y-intersect = 151.0). Error bars show the standard error of the mean (SEM).

Sequential antidromic FETi spikes evoke EPSPs of diminishing amplitude in flexors (Figs 2A,B, S2A–C) demonstrating STD at this synapse^10^. In contrast to many other weak synapses, STD can be observed at the FETi-flexor synapse without requiring any averaging (Fig. 2A)^6,10,11^. We assessed STD at the FETi-flexor synapse with sequences of 10 spikes over a range of frequencies from 5 to 30 Hz (Figs 2A–C, S2C). During each sequence, depression was greatest between the first and second spikes, thereafter reaching a steady-state where little further depression occurred (Figs 2A–C, S2C). The steady-state amplitude attained after 10 spikes was dependent upon the presynaptic spike frequency (Fig. 2C). At 5 Hz the steady-state EPSP amplitude was ∼58% of the initial EPSP amplitude, whereas at 10 Hz the EPSP amplitude was ∼39%; at 15 Hz ∼27%, and at 30 Hz just ∼6% (Fig. 2C; Table 1). The steady-state EPSP amplitude was significantly different among the frequencies we tested (F_3,52_ = 323.6, p < 0.001, N = 16; one-way ANOVA), demonstrating that STD is frequency-dependent at the FETi-flexor synapse^6^.

**Table 1:** EPSP relative amplitudes at different time points.

Frequency (Hz)	Amplitude (%)		
	10^th^ EPSP (N = 16)	200 ms recovery (N = 13)	1600 ms recovery (N = 13)
5	58.57 ± 1.07	57.93 ± 1.33	84.54 ± 2.71
10	39.48 ± 1.09	55.35 ± 2.11	82.42 ± 1.71
15	27.72 ± 1.96	54.77 ± 1.61	83.97 ± 1.44
30	6.40 ± 0.38	56.55 ± 2.68	85.98 ± 2.89

Relative amplitude of the final flexor EPSP evoked by the FETi spike train and the flexor EPSP amplitudes at the 200 ms and 1600 ms recovery points.

We assessed the recovery of EPSP amplitude at the FETi-flexor synapse with single antidromic spikes between 200 and 1600 ms after each train of 10 spikes (Figs 2A,B,D, S2A,B,D). Irrespective of the frequency of the preceding train of 10 spikes the amplitude of the flexor EPSP recovered to ∼55–60% of its initial amplitude after 200 ms (Figs 2D, S2D; Table 1). Indeed, there was no significant difference in the amplitude of the flexor EPSP after 200 ms of recovery among all of the different 10 spike train stimulus frequencies (F_3,44_ = 0.5851, p > 0.05, N = 13; one-way ANOVA). For each of the other recovery durations the EPSP amplitude always recovered to the same amplitude irrespective of the frequency of the spikes in the stimulus and, therefore, the extent of the depression (Fig. 2D, S2D; Table 1). Therefore, the amplitude of the EPSP after recovery is independent of the extent of depression.

Most of the recovery occurred within the first 600 ms, after which the EPSP amplitude continued to recover but at a slower rate reaching ∼80–85% of its initial amplitude after 1600 ms (Fig. 2D). Again, this recovery was independent of the frequency of the preceding 10 spikes (F_3,32_ = 0.403, p > 0.05, N = 10; one-way ANOVA). Consequently, there was also no correlation between the steady-state EPSP amplitude and the recovery after 200 (r_47_ = 0.250, p > 0.05), 400 (r_45_ = 0.248, p > 0.05) and 600 ms (r_47_ = 0.075, p > 0.05). Thus, at the FETi-flexor synapse recovery, like STD, is frequency-dependent with faster recovery following greater depression. Such frequency-dependent recovery has been demonstrated at both the Calyx of Held and at the FETi-flexor synapse^5,6^.

### Matched Rates of Short-Term Depression and Recovery

The rate at which the FETi-flexor synapse depressed increased with the frequency of antidromic stimulation (Fig. 2C). We quantified this change in STD dynamics by fitting an exponential with a single time constant to the EPSP amplitude to yield a time constant of depression, τ_dep_ (Fig. 2C). τ_dep_ was faster at higher stimulation frequencies, decreasing from ∼275 ms at 5 Hz to ∼15 ms at 30 Hz (Table S1). τ_dep_ was significantly different across all stimulation frequencies (H_3,13_ = 36.56, p < 0.001; Kruskal-Wallis test with Dunn’s post-hoc test) (Fig. 2C,E). We also analyzed the rate of recovery in the same way, obtaining a recovery time constant, τ_rec_ (Fig. 2D,E). τ_rec_ was also faster following higher stimulation frequencies, decreasing from ∼916 ms at 5 Hz to ∼193 ms at 30 Hz (Table S1). Again, τ_rec_ was significantly different across all stimulation frequencies (H_3,13_ = 2.85, p < 0.001, Kruskal-Wallis test with Dunn’s post-hoc test) (Fig. 2D,E). Thus, both τ_dep_ and τ_rec_ are frequency dependent. Comparison of τ_dep_ with τ_rec_ showed that at the lowest spike frequency the rate of depression and recovery were the slowest, and as the stimulation frequency increased so too did the rates of depression and recovery (Fig. 2E). Thus, τ_dep_ and τ_rec_ were strongly correlated in all our recordings (r_3_ = 0.97, p < 0.05; Pearson correlation) (Fig. 2F) so that the rate and extent of STD is matched to the rate and extent of recovery at each frequency.

The soma of each flexor motor neuron is not interposed between the dendrites and the primary neurite where spikes are initiated, and instead is linked to the rest of the neuron through a small branch (Fig. 1B–D)^10,11,14^. Short-term depression has been recorded at the FETi-flexor synapse^10^. Nevertheless, it is important to ensure that the electrical signals recorded intracellularly at the soma are representative of those signals in the dendrites and primary neurite where computation occurs. We modelled the FETi motor neuron and a flexor, as well as the FETi-flexor synapse. Our model consisted of a single presynaptic compartment, representing FETi, and a multi-compartment model of a flexor with compartments representing the soma, dendrites, primary neurite, spike initiation zone and axon (Fig. S3A). The FETi compartment was coupled to the multi-compartment flexor model by a depressing synapse.

We assessed the EPSPs produced in the soma of the multi-compartment flexor model to sequences of 10 spikes (Fig. S3B). These models closely reproduced the amplitudes of the EPSPs recorded intracellularly from the flexor soma at frequencies from 5 to 30 Hz (Fig. S3C). We assessed the amplitudes of the EPSPs in the dendrites and compared them to those in the flexor soma (Fig. S3B). The amplitudes of the dendritic EPSPs from the flexor model followed the same trends as those of soma (Fig. S3C). We fitted time constants to both the dendritic EPSPs and the soma EPSPs (Fig. S3C) and compared these time constants to those fitted to experimental EPSP amplitudes (Fig. S3D). The time constants obtained from fits to the soma or dendrites were similar, though those fitted to dendritic EPSPs amplitudes were faster at lower frequencies (Fig. S3D). However, the time constants obtained from both the dendritic and somatic EPSPs maintained a linear relationship with the time constants obtained from experimental EPSPs, showing that the dynamics we observed in the soma were indeed accurate.

### EPSP Amplitude Encodes Interspike Interval (ISI)

**Figure 3:**
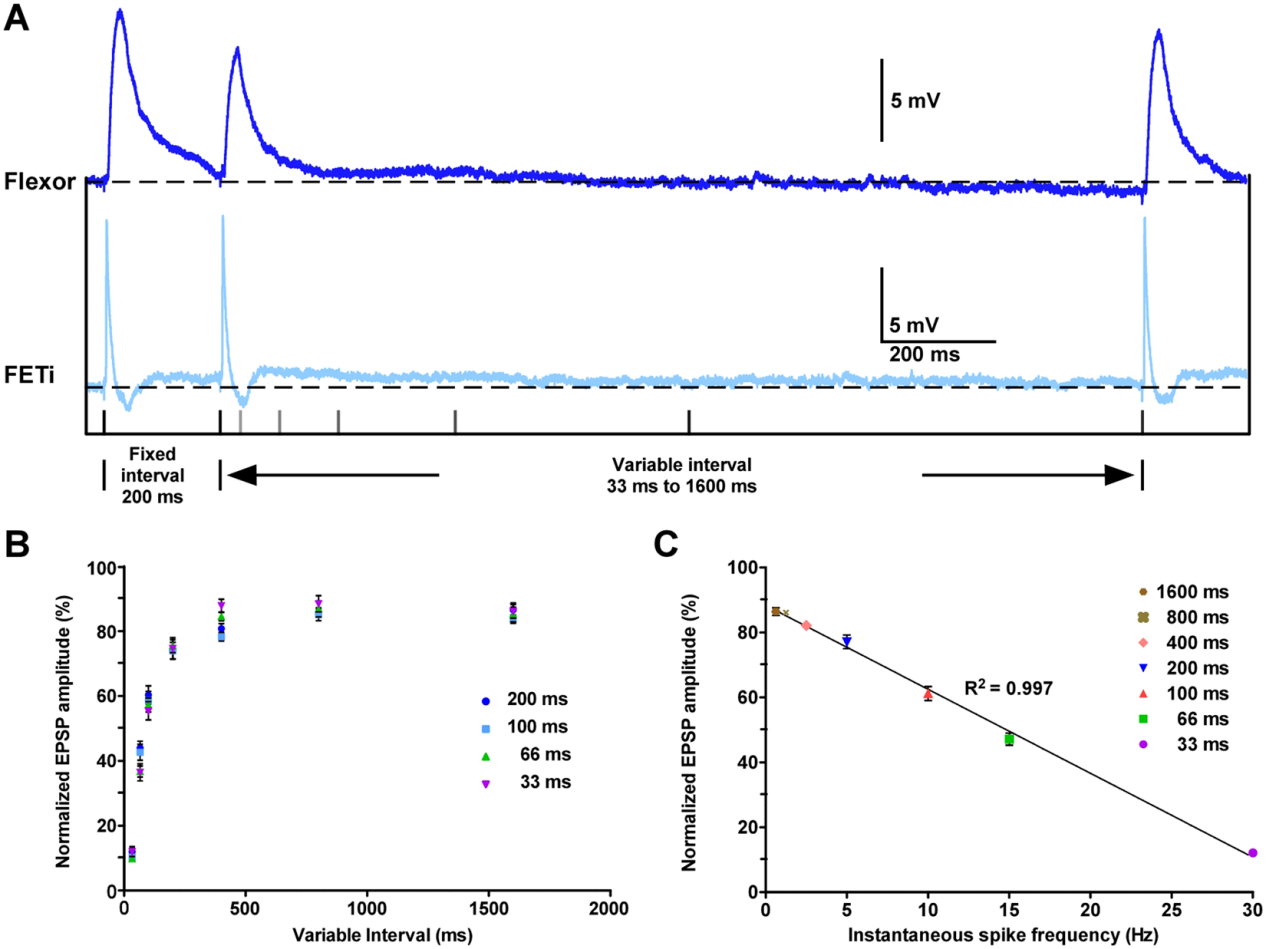
The FETi-flexor EPSP amplitude encodes interspike interval. (**A**) Intracellular traces showing flexor (dark blue) EPSPs evoked by triplets of FETi (pale blue) antidromic spikes. The stimulation protocol is shown below. (**B**) The normalized flexor EPSP amplitudes at each of the different variable intervals from the stimulation protocol shown in A. Four different fixed intervals were used 33, 66, 100 and 200 ms. The normalized EPSPs amplitude is the same irrespective of the duration of the fixed interval. Data were obtained with 3 repeats of each stimulus in 8 animals. (**C**) Mean normalized EPSP amplitudes at different variable intervals grouped by the instantaneous spike frequency (ISF). Flexor EPSP amplitude is related to FETi ISF, decreasing linearly as the ISF increases (slope = −2.59, Y intersect = 88.39). Data were obtained with 3 repeats of each stimulus in 8 animals.

We determined the relationship between EPSP amplitude and ISI using triplets of spikes, which is equivalent to a paired-pulse stimulus with a third recovery pulse. Intervals between the first and second spikes of 33, 66, 100 and 200 ms were each followed by an interval from 33 to 1600 ms between the second and third spikes (Fig. 3A). For example, following a first interval of 200 ms (Fig. 3A) the EPSP amplitude after a second interval of 33 ms was ∼11%, rising to ∼44% after 66 ms, and ∼86% after 1600 ms (Fig. 3B; Table S2). Similar EPSP amplitudes were obtained for second intervals of a particular duration, irrespective of the duration of the first intervals (Fig. 3B). We fitted a single exponential to the EPSP amplitudes following a particular first interval (Table S2). There was no significant difference among the time constants (H_3,8_ = 5.27, p > 0.001; Kruskal-Wallis test with Dunn’s post-hoc test), confirming that EPSP amplitude at this synapse is dependent only upon the most recent ISI (i.e. the second interval) and is independent of previous activity (i.e. the first interval) (Fig. 3B).

The reciprocal of the FETi ISI, the instantaneous spiking frequency (ISF), is encoded linearly by the relative amplitude of the flexor EPSP over behaviorally relevant frequencies (Fig. 3C). The EPSP amplitude was significantly different across ISFs (F_6,48_ = 341.4, p < 0.001; one way ANOVA with Bonferroni post-hoc test), except at very low spike frequencies (0.625 and 1.25 Hz), which were not significantly different from one another (Fig. 3C).

### Matching of STD and Recovery is Necessary

We used a phenomenological computational model to assess the implications of matched depression and recovery dynamics on information coding at the FETi-flexor synapse (see Materials and methods)^11,15–17^. The model uses the timing of FETi spikes to estimate flexor EPSP amplitude from the absolute response if all transmitter at the synapse was released, *A*, the fraction of available transmitter used after a spike, *U*, and the time constant of recovery between spikes, τ_Rec_.

**Figure 4:**
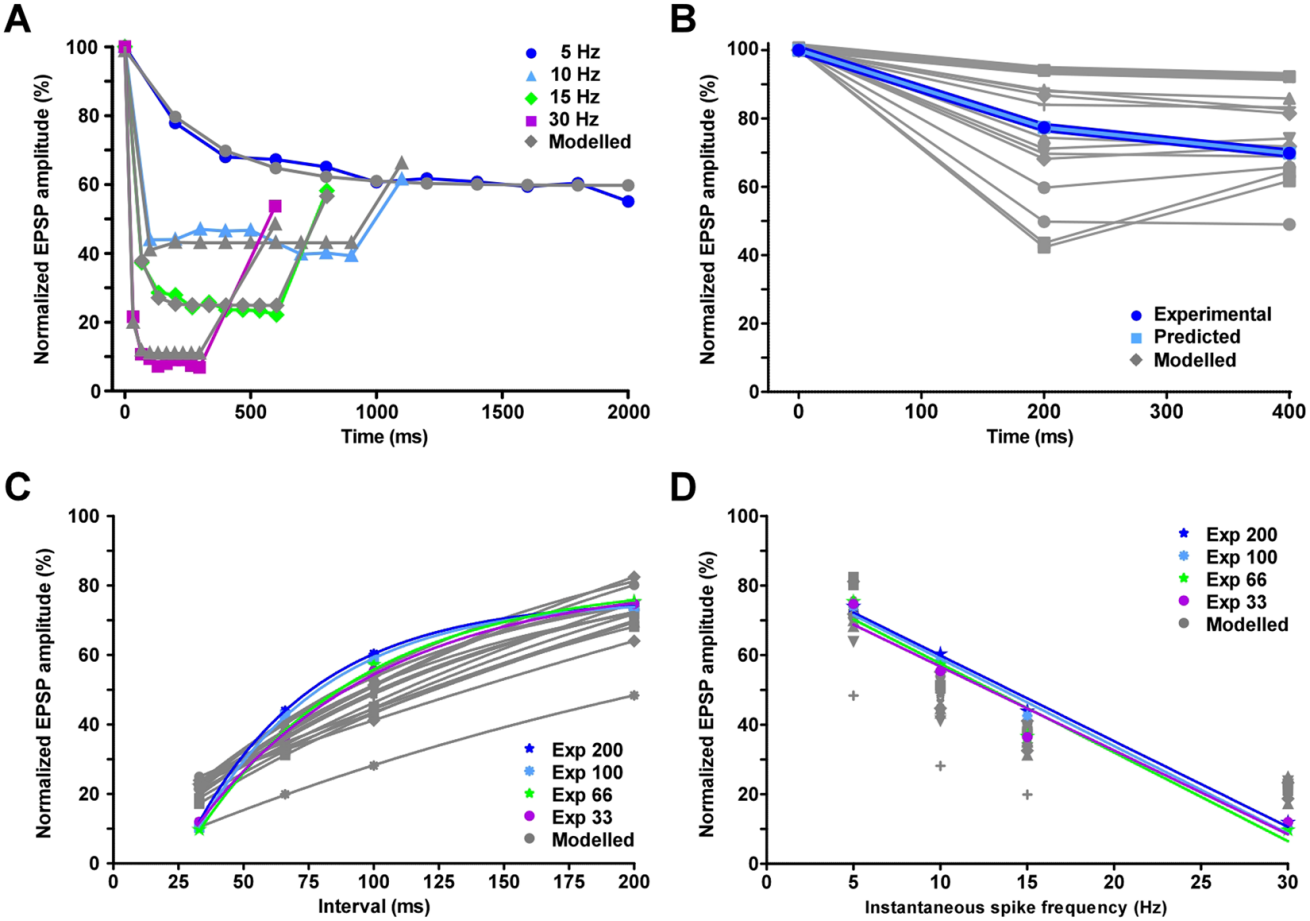
Computational modelling of the FETi-flexor synapse demonstrates the importance of matching the frequency-dependent time constants of depression and recovery. (**A**) The fits of the computational model to trains of spikes causing STD in flexor EPSP amplitude. EPSPs evoked by spikes at different frequencies were fitted independently (see Supplemental Experimental Procedures). The computational model fits both the STD and recovery. Model parameter at 5 Hz, U = 0.64; A = 153.68; τ_rec_ = 566 ms. At 10 Hz, U = 1.02; A = 96.74; τ_rec_ = 166 ms. At 15 Hz, U = 0.86; A = 115.81; τ_rec_ = 290 ms and at 30 Hz, U = 0.91; A = 110.74; τ_rec_ = 322 ms. (**B**) The computational model fits experimental flexor EPSP amplitudes evoked by spike triplets. Flexor EPSP amplitudes are shown for a single 200/200 ms interval combination. Replacing the recovery time constant for this 200/200 ms (fixed/variable) interval combination with time constants obtained from fits of other intervals prevents the model fitting the experimental data. The dark blue line represents the experimental data. The pale blue data represent the model fit. Model parameter: U = 0.81; A = 122.77; τ_rec_ = 344 ms. (**C**) The relationship between normalized flexor EPSP amplitude and interspike interval is predicted by the model. Replacing the recovery time constant for all four fixed intervals and variable intervals of 33, 66, 100, 200 ms with time constants obtained from fits of other intervals, alters the relationship. Colored lines represent the experimental data, while grey lines represent the modeled data. (**D**) The relationship between normalized Flexor EPSP amplitude and ISF is predicted by the model. Replacing the recovery time constant for all four fixed intervals and variable intervals of 33, 66, 100, 200 ms with time constants obtained from fits of other intervals, disrupts the linear relationship between flexor EPSP amplitude and ISF. Colored lines represent the experimental data while grey lines represent the modeled data.

We fitted the computational model to the sequences of 10 FETi spikes and corresponding flexor EPSP amplitudes. At any given spike frequency the model fitted the flexor EPSP amplitudes (Fig. 4A). We used the fitted parameters to predict flexor EPSP amplitudes evoked by other spike frequencies. However, parameters that accurately fitted EPSP amplitudes at one frequency could not predict EPSP amplitudes measured at any other frequency with similar accuracy (Table S3). The error between the observed and fitted EPSP amplitudes ranged from 14.68 to 30.79 mV (see Materials and methods), whereas the error between the observed and predicted EPSP amplitudes increased from 41.80 to 135.17 mV (Table S3). This is consistent with the frequency-dependency of STD and recovery at the FETi-flexor synapse. A similar inability of parameters fitted to one frequency to predict parameters at other frequencies has been observed previously^11,18^.

The model was also fitted to the experimental data from the triplet spike paradigm (Fig. 4B). Again, the model fitted the EPSP amplitudes evoked by any given combination of first and second interval but was unable to predict the EPSP amplitudes of another set of intervals accurately (Figs 4B, S4A–D). The resulting EPSP amplitudes predicted by the parameters for a given interval are either under or overestimated (Fig. 4C); the models consistently predicted EPSP amplitudes that were too high below 33 ms but lower than those observed experimentally between 33 and 150 ms. The lower EPSP amplitudes are the consequence of reduced recovery, which causes a mismatch between depression and recovery dynamics. Consequently, the linear encoding of the FETi ISF in the flexor EPSP amplitude is disrupted (Fig. 4D). Thus, without matched dynamics of depression and recovery, the synapse was incapable of linear encoding of the FETi ISF.

### Matched Dynamics Occur in Naturalistic Sequences

**Figure 5:**
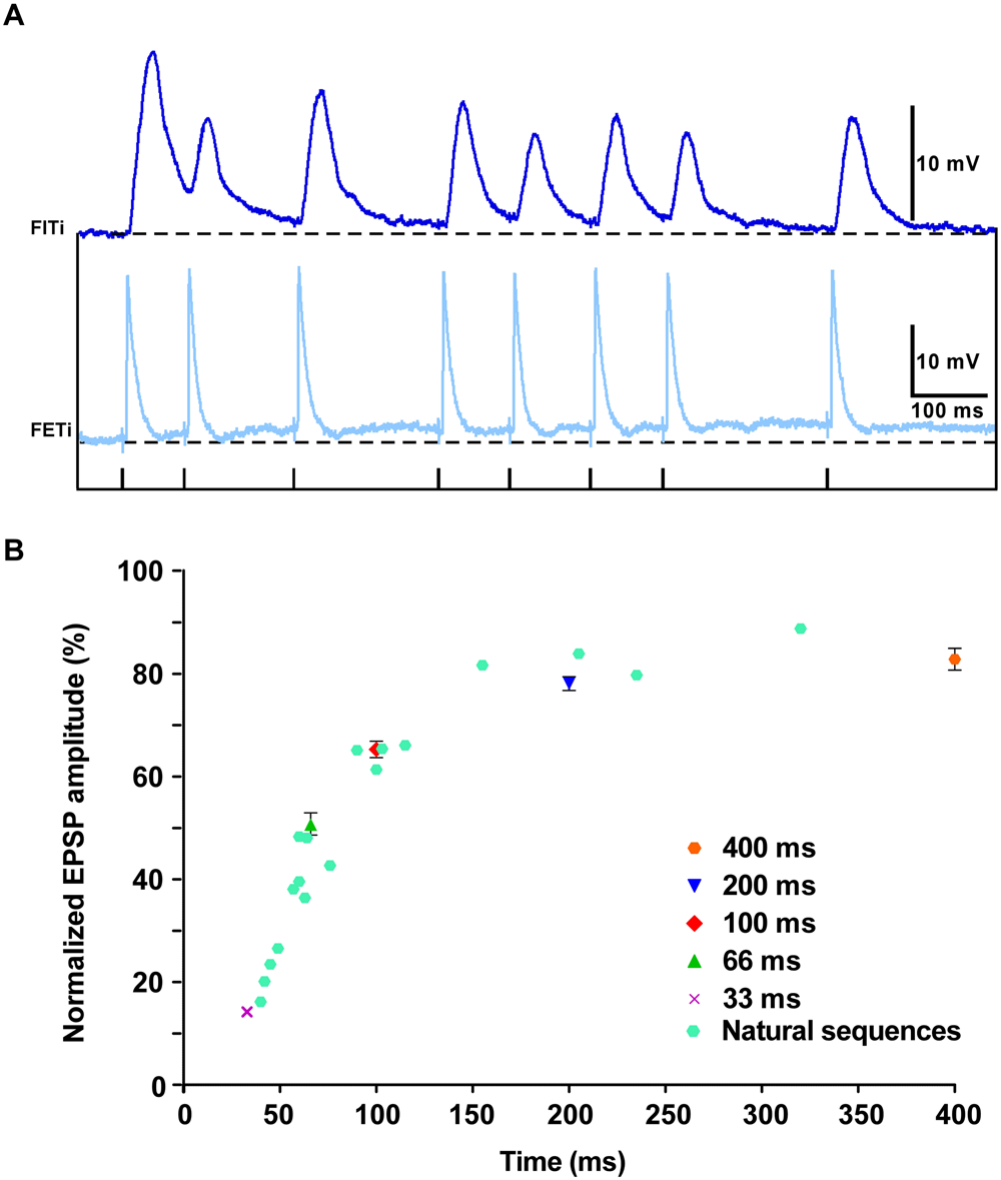
The FETi-flexor EPSP amplitude during natural sequences encodes interspike interval. (**A**) Intracellular traces showing flexor EPSPs evoked by natural sequences of FETi antidromic spikes. The stimulation protocol is shown below. (**B**) The amplitudes of flexor EPSPs during natural sequences have the same relationship to the inter-spike interval as do triplets (see Fig. 3).

We made paired recordings of FETi and a flexor during naturalistic hind leg movements (Fig. 5A). Such movements included jumping and kicking as well as flicking, a previously undescribed movement. The interspike interval (ISI) during these sequences differed depending on the specific behavior. During flicks the ISI was 45.6 ± 34 ms (mean ± SD), which was lower 32.9 ± 17 ms for kicks. Sequences of activity during these behaviours were recorded and then sequences of spikes were played back to the FETi motor neuron (Fig. 5A). In this way, the amplitudes of the flexor EPSPs could be measured in isolation, without other synaptic inputs related to the production of the behaviours. The amplitudes of the flexor EPSPs during these playback sequences were measured and related to FETi ISIs. We compared the flexor EPSP amplitudes from a range of natural ISIs to those obtained from constant frequency stimuli (Fig. 5B). Over the entire range of ISIs generated during natural sequences, the coding remains as predicted by the constant frequency modelling, emphasizing that the flexor EPSP amplitude does indeed code FETi ISI during behaviour.

## Discussion

The dependence of EPSP amplitude upon ISI duration (or the reciprocal, ISF) allows the FETi-flexor synapse to encode spike timing over a range of intervals (or frequencies) that occur during natural behavior *in vivo*. Encoding ISI duration depends upon three features of the synapse: (1) frequency dependent short-term depression (STD) and recovery; (2) matching of the time constants of frequency dependency of STD and recovery; and (3) low variability in the EPSP amplitude at a given ISI. Without the precise matching of the depression and recovery time constants this coding scheme would fail because deviations introduce non-linearities so that EPSP amplitudes no longer correspond to specific ISIs. The low variability in EPSP amplitudes evoked by a given pre-synaptic ISI is also crucial because substantial variability would obscure the relationship between EPSP amplitude and the pre-synaptic ISI.

We used antidromic stimulation to generate the presynaptic spike trains on which are analysis of the FETi-flexor synapse depends. Antidromic spikes are unlikely to differ from orthodromic spikes evoked by current injection into the FETi soma. However, differences have been demonstrated between spikes evoked by antidromic stimuli and those evoked by synaptic inputs^11^. These synaptic inputs to FETi, which occur close to or on the same dendritic branches that contain the output synapse to the flexors, can alter the spike shape thereby modifying the strength of the FETi-flexor synapse^11^. Thus, the depression and recovery dynamics of the FETi-flexor synapse will be modified during natural behaviours evoked by synaptic inputs to FETi, though how this modifies the coding of ISI is not clear from the analysis presented here.

Many central synapses show STD^19^, and the frequency-dependency of recovery has also been described at both the Calyx of Held and the FETi-flexor synapse^5,6^. Yet to our knowledge, no other synapse has been shown to encode ISI duration in EPSP amplitude. One reason may be that the FETi-flexor synapse has several properties that make it atypical. Firstly, it is highly reliable producing low variability EPSPs as much as 27 mV in amplitude as measured in the soma^10,13^. It is the only monosynaptic chemical connection between motor neurons that has been documented in the insects, formed between the dendritic branches of the metathoracic FETi and flexors making a dendro-dendritic synapse at which input and output synapses are in close apposition^20–22^.

Yet these features do not preclude similar coding mechanisms existing at other synapses. The FETi-flexor synapse is glutamatergic^23^, and many molecular components may have been co-opted from the neuromuscular junction, which in insects is glutamatergic^24^. There is no evidence to suggest that locusts possess classes of synaptic proteins not also found at other synapses in vertebrates and invertebrates alike, though vertebrates do possess synaptic components not found in invertebrates^25^. Indeed, many components assembled at synapses may have been co-opted from other cellular functions and were present early in the evolution of the animals^26–28^. Thus, there appears to be no biological constraint preventing other synapses from producing similar dynamics.

Coding strategies similar to those of the FETi-flexor synapse may not have been observed before because few studies have quantified recovery and such dynamics may be difficult to observe at weak synaptic connections given that it is necessary to analyze highly averaged datasets, a process that never happens *in vivo*.

Of those synapses at which recovery dynamics have been quantified, excitatory neurons in layers V and IV of rat somatosensory cortex^29^ and both the End Bulb and Calyx of Held synapses also show frequency-dependent recovery^5,7^. However, the depression and recovery time constants at these synapses are not matched. The rate of vesicle recycling may prevent frequency dependent recovery being matched to STD at the Calyx. It is also important to recognize that these synapses are embedded within different circuits and are involved in different computations. There is still much discussion and reinterpretation of the mechanisms responsible for the dynamics of STD and recovery in these synapses because many of these preparations involve recording STD *in vitro* and often from immature synapses^30–33^ making it difficult to interpret the dynamics and the mechanisms underpinning STD *in vivo* within a behavioral context.

Short-term depression is thought to perform a variety of tasks in numerous neural circuits^19,34,35^, mediating information transfer in cortical neurons^1,3,15^, temporal filtering in electric fish^36^, contrast adaptation in visual systems^37^, and in mammalian and avian auditory brainstems^5,7,38–40^ where STD is thought to play critical role on the computations of sound processing. The computational role of STD in these systems has often been interpreted in terms of the changes evoked in the steady-state EPSP amplitude. However, in many circuits neural computations must occur within a few spikes to operate on behaviorally relevant time scales, precluding time averaging. Our results show that STD and recovery can form a key part of spike time dependent coding in neural circuits. However, STD may take on a variety of roles depending on the precise context, being involved in spike timing at some synapses and spike rate at others.

## Materials and Methods

### Animal preparation and electrophysiology

Adult desert locusts (*Schistocerca gregaria*, Forskål, 1775) were taken from a crowded colony maintained at the Department of Zoology, University of Cambridge, UK. Locusts were mounted ventral side uppermost in a platform of modelling clay (Plasticine®). The pro- and mesothoracic legs and the femur and tibia of one hind leg were fully restrained. The tibia of the opposite hind leg was free to move. A pair of 50 µm wires insulated except for their tip was inserted into the extensor tibia muscle and another pair into the flexor tibia muscle of this hind leg to record the muscle activity. A window was cut in the ventral thorax to permit access to the metathoracic ganglion. The air sacs surrounding the metathoracic ganglion were removed leaving the main trachea intact. The meso- and metathoracic ganglia were stabilized on a custom-made wax-coated silver platform and then pinned down to prevent movement during the experiments.

The preparation was perfused continuously with physiological saline consisting of 140 mM NaCl, 10 mM KCl, 4 mM CaCl_2_, 4 mM NaHCO_3_, 6 mM NaHPO_4_^41^ at room temperature (22–25 °C). The ventral nerve cord connectives and peripheral nerves were left intact. The sheath of the ganglion was treated with direct application of protease (Sigma type XIV) for 45 to 60 seconds^11^ to allow penetration with thick-walled glass microelectrodes (50 to 70 MΩ) filled with 2 M potassium acetate.

Paired intracellular recordings were made from the fast extensor tibiae motor neuron (FETi) and flexor tibiae motor neurons (flexors) that control the extension and flexion of the tibia of the hind leg, respectively. The FETi motor neuron was identified by electrical stimulation of its peripheral axon terminals in the extensor muscle hind leg, to evoke an antidromic spike that can be recorded from the soma. The flexors were identified by a short latency monosynaptic EPSP after each antidromic spike in the FETi motor neuron^10,42^. Individual flexors were identified by the positions of their somata relative to that of the FETi motor neuron and the other flexors; by their responses to antidromic stimulation of the FETi motor neuron, the EPSP eliciting more spikes in slow flexors than in the fast flexors; by their responses to extension of the ipsilateral metathoracic tibia; and by correlation of their activity with the movements of the tibia in response to stimulation of other legs and body parts^6^. Intracellular recordings were made from the FETi motor neuron and one flexor.

To study the dynamics of the FETi-flexor synapse in isolation, all legs were restrained and the distal femur of the ipsilateral leg was cut to prevent inputs from the Femoral Chordotonal Organ reaching the motor neurons^43^. The connectives linking the mesothoracic ganglion to the prothoracic ganglion, those linking the metathoracic ganglion to the fourth abdominal ganglion and all peripheral nerves on both sides were cut except nerve 5. This prevented any descending, ascending and peripheral inputs from altering the dynamics of the FETi-flexor synapse but still allowed the identification of the motor neurons *via* antidromic spikes from muscle stimulation^6,11,44^.

### Stimulation protocol

Antidromic stimulation of the FETi motor neuron was used to trigger action potentials for all of the stimulation regimes. By cutting all the peripheral nerves except nerve 5, we ensured that simulation only evoked an antidromic spike in the FETi motor neuron. This permits precise control of the timing of the action potential and avoids the need to inject large currents into the FETi motor neuron soma to trigger action potentials at the spike initiation zone. It also ensures that the action potentials are not triggered by synaptic inputs, which in the FETi motor neuron are known to shunt the action potential, altering synaptic strength^11^. The FETi motor neuron is the only one in the desert locust known to have a central output synapse^10^, so that EPSPs occurring in flexor motor neurons with a short latency after an antidromic spike in FETi can only be the result of this synapse. Moreover, the latency between a spike in FETi and an EPSP in a flexor is so short that it can only be generated by a monosynaptic connection^6,10,11^.

A train of 10 antidromic FETi motor neuron spikes at frequencies of 5, 10, 15 and 30 Hz was generated to induce short-term synaptic depression. Each stimulus train was followed by a single stimulus at an interval ranging from 200 to 1600 ms to quantify the recovery of the EPSP. A resting period of 45 to 60 seconds was given after every recovery pulse before the next spike train to give the synapse time to recover fully.

A train of three antidromic FETi motor neuron spikes was generated to study the effect of the ISF on the relative amplitude of the EPSP. The interval between the first and the second spike was fixed. Each stimulus pair was followed by a single stimulus at an interval of 33 to 1600 ms. This protocol was repeated for fixed intervals of 33, 66, 100 and 200 ms. A period of 20 seconds was given between sequences of stimulus.

Natural sequences of FETi motor neuron spikes during flicking movements of the hind leg were recorded and ‘played back’ to the FETi-flexor synapse as trains of stimuli that generated antidromic spikes. These sequences contained interspike intervals that correspond exactly to those of natural sequences of activity. Four distinct natural sequences were used for these playback experiments.

### Data analysis

Intracellular recordings were A/D converted on-line using a CED1401 acquisition board and Spike 2 software (Cambridge Electronic Design, Cambridge, UK) and stored for subsequent offline analysis.

The amplitudes of flexor EPSPs were normalized by dividing by the amplitude of the EPSP evoked by the first FETi spike within a train and multiplying it by a hundred. This procedure was applied to all the EPSPs within a given spike train including the specific recovery pulse for that train. Values are given as mean ± SE; N refers to the number of animals and n to the number of repeats per animal. Overall differences between stimulation frequencies were determined by one-way ANOVA where F_df,df_ represents the F-statistic and its degrees of freedom^45^.

For each individual cell, the data for depression and recovery was fitted with a single exponential to reveal the time constant of depression τ_dep_ and time constant of recovery τ_rec_ for that cell. The τ_dep_ and τ_rec_ of each cell were then pooled to obtain the population average τ_dep_ and τ_rec._ Overall differences in τ_dep_ and τ_rec_ between stimulation frequencies were determined by Kruskal-Wallis Test (H test) with Dunn’s post hoc analysis, were H_df,df_ represents the H-statistic and its degrees of freedom^45^.

To measure of the strength and direction of the linear relationship between τ_dep_ and τ_rec_ we used Pearson correlation, were r_df_ represents Pearson’s r statistic and its degrees of freedom^45^.

All statistical tests were performed using Microsoft Excel 2007 (Microsoft, Redmond, USA) and Prism 5.02 (GraphPad Software, San Diego, California USA).

### Multi-compartment modelling

To determine whether activity in the soma of the flexor tibiae motor neurons reflects activity within the dendritic branches close to synaptic inputs, we modelled the FETi motor neuron as a single pre-synaptic electrical compartment and the flexor motor neuron as a five electrical compartment model. Our five compartment flexor model was based upon a three compartment model created by Peron and Gabbiani^46^. The five electrical compartments of the flexor motor model represent the dendrites, soma and axon, as well as a primary neurite and the spike initiation zone (Fig. S3A). The primary neurite was linked to the dendrites and spike initiation zone, as well as the soma, whereas the spike initiation zone was linked to the primary neurite and dendrites, as well as the axon. Each compartment, both pre- and postsynaptic, had specific membrane capacitance, C_m_, of 1 μF/cm^2^ and a leak current:

1 $${I}_{L}={g}_{L}(V-{E}_{L})$$

The leak conductance, g_L_, had a value of 0.1 mS/cm^2^ with an equilibrium potential, E_L_, of −75 mV. Each compartment of the flexor motor neuron model was connected using connection currents:

2 $${I}_{C}=\frac{{g}_{C}}{{a}_{rel}}(V-{V}_{C})$$

where g_C_ is the maximal compartmental conductance 0.13 mS/cm^2^, V_C_ is the voltage of the connected compartment, and a_rel_ is the size of the given chamber, a_1_, relative to the size of the two connected compartments, a_1_ + a_2_. Compartment sizes were estimated from camera lucida drawings^47^. The dendrites had an area of 50,425 um^2^, the spike initiation zone 6,134 um^2^, the axon 5,152 um^2^, the primary neurite 3,557 um^2^, and the soma 4,452 um^2^.

The dendrites, primary neurite and soma of the flexor motor neuron were modelled as passive compartments with a rectifier current:

3 $${I}_{R}={g}_{R}r(V-{E}_{R})$$

(g_R_ = 0.5 mS/cm^2^, E_R_ = −35 mV). The gating variable, r, followed first-order kinetics

4 $$\frac{dx}{dt}={\alpha }_{x}(V)(1-x)-{\beta }_{x}(V)x$$

with α_R_ = 0.0018 exp[(V + 86.6)/47.5], β_R_ = 0.97 exp[(V + 85.2)/17.7]/6. The membrane potentials of the passive compartments were regulated by the general equation:

5 $${C}_{m}\frac{d{V}_{pass}}{dt}=-\,{I}_{L}-{I}_{R}-{I}_{C}$$

The spike initiation zone of the FETi motor neuron and the axon of FlTi were modelled as active compartments with a Na^+^ current

6 $${I}_{Na}={g}_{Na}{m}^{3}h(V-{E}_{Na})$$

and a K^+^ current

7 $${I}_{K}={g}_{K}{n}^{4}(V-{E}_{K})$$

(g_Na_ = 30 mS/cm^2^, E_Na_ = 40 mV, g_K_ = 10 mS/cm^2^, E_K_ = −75 mV). Gating variables *m*, *h* and *n* followed first-order kinetics (4). α_m_ = −(V + 33)/{[exp(−0.1 * (V + 33)) − 1] *  5.65}, β_m_ = 5 exp[−(V + 54)/12], α_h_ = 0.1 exp[−(V + 58)/10], β_h_ = 5/{4 * [exp(−0.1 * (V + 30)) + 1]}, α_n_ = −(V + 34)/27 * {exp[−0.1 * (V + 34)] − 1}, β_n_ = 3.75 * exp[−(V + 44)/25]/7.4. The membrane potentials of the active compartments were therefore regulated by the general equation:

8 $${C}_{m}\frac{d{V}_{act}}{dt}=-\,{I}_{L}-{I}_{Na}-{I}_{K}-{I}_{C}$$

The FETi was modelled as a single compartmental neuron and so it had no connection current, I_C_. Current injection into the FETi compartment of 1.46165 μA/cm^2^ produced 5 Hz responses in FlTi, injection of 1.4857 μA/cm^2^ produced 10 Hz spikes, 1.5175 μA/cm^2^ produced 15 Hz spikes, and 1.648 μA/cm^2^ produced 30 Hz spikes. The dendritic compartment of FlTi had a fast glutamatergic synapse with current

9 $${I}_{S}={g}_{S}s(V-{E}_{S})$$

(g_S_ = 100 mS/cm^2^, E_S_ = 40 mV). The glutamatergic channel opening probability, s, also followed first order kinetics (4). Its closing rate β_s_ was constant at 35 s^−1^, while α_s_ was a binary variable^48^; 0 during resting states and 0.25 s^−1^ when FETi had voltage values of −45 mV or above.

To implement short term depression (STD), s was modulated at each integration by a depressing factor f_D_, which represents the fraction of neurotransmitter available. The maximum and initial value of available neurotransmitter was f_Dmax_ = 1. The available neurotransmitter was kept at 100% for 190 ms (5 Hz STD model), 104 ms (10 Hz STD model), 69 ms (15 Hz STD model), and 50 ms (30 Hz STD model), to ensure the full delivery of the first FlTi spike. After these time limits, f_D_ started decreasing when FETi voltage values were above voltage limits of −10 mV (5 Hz STD model), −30 mV (10 Hz STD model), −50 mV (15 Hz STD model) and −55 mV (30 Hz STD model). Decrease neurotransmitter rates were 99.9955% (5 Hz STD model), 99.9835% (10 Hz STD model), 99.9892% (15 Hz STD model), and 99.978% (30 Hz STD model). When the FETi membrane voltage was lower than the voltage limits, f_D_ recovered following

10 $$\frac{d{f}_{D}}{dt}=\frac{{f}_{Dmax}-\,{f}_{D}}{{\tau }_{D}}$$

The recovery time constant, τ_D_, was 778 for the 5 Hz STD model, 99 for the 10 Hz STD model, 75 for the 15 Hz STD model, and 28 for the 30 Hz STD model.

Constants of the active compartments were adjusted by tuning our FETi single compartment model to axonal intracellular recordings from the FETi motor neuron^14^. Constants of the passive compartments and synaptic transmission were adjusted by tuning our FlTi soma model to our own somatic intracellular recordings. The STD dynamics were also tuned to our experimental values for three of the STD frequencies we tested.

All integration was carried out using explicit Euler integration (time step, h, is 0.05 ms):

11 $${q}_{t}={q}_{t-1}+h\frac{dq}{dt}$$

### Synaptic depression modelling

A three-parameter computational model was used to characterize STD and recovery^11,16,17^. The parameters are the absolute response if all transmitter at the synapse was released, *A*, the fraction of available transmitter used after a spike, *U*, and the time course of recovery between spikes, τ_Rec_. Each spike releases a fraction (*U*) of the absolute available transmitter (*A*) evoking an EPSP. The released transmitter, R, recovers with a time constant, τ_Rec_. For the first spike A*U determines the amount of transmitter released. The remaining transmitter plus the amount of transmitter recovered is available at the next spike. Hence, the released transmitter depends upon the timing of subsequent spikes:

$${R}_{n+1}={R}_{n}(1-U)\exp \,(\frac{-{\rm{\Delta }}t}{{\tau }_{Rec}})+1-\exp (\frac{-{\rm{\Delta }}t}{{\tau }_{Rec}})$$

where Δt is the interval between the nth and (n + 1)th spike.

Experimental data were fitted by iterating the parameters (*A, U*, and τ_Rec_) of the basic model and minimizing (*EPSP*_*Experiment*_ − *EPSP*_*Model*_)^2^ for all EPSPs in a spike train. The starting parameters (*A, U*, and τ_Rec_) were chosen at random, and the model was iterated to produce a minimum. The fit of the model to the data and its ability to predict novel spike trains was assessed by comparing the standard error of the estimate divided by the mean^49^. The total error of the fitting was obtained by adding the product obtained from the calculation of Euclidean distance between the experimental EPSP value and the models predicted EPSP values for each time point:

$$Total\,error=\sum \surd {(EPS{P}_{exp}-EPS{P}_{model})}^{2}$$

## Electronic supplementary material

Supplementary Material

## Data Availability

The datasets generated during and/or analyzed during the current study are available within the Supplemental Information and the raw data are available from the corresponding author.
