# Supplementary material for: Matched Short-Term Depression and Recovery Encodes Interspike Interval at a Central Synapse
Source: Sci Rep. 2018 Sep 11;8:13629. doi: 10.1038/s41598-018-31996-0 (PMC6134063; doi:10.1038/s41598-018-31996-0)
Supplement: Supplementary file 1 — Supplementary Material [file 41598_2018_31996_MOESM1_ESM.pdf]

Supplemental Information: Matched Short-Term Depression and Recovery Encodes Interspike Interval at a Central Synapse

Armando E. Castillo, Sergio Rossoni and Jeremy E. Niven

Supplemental Figure and Tables

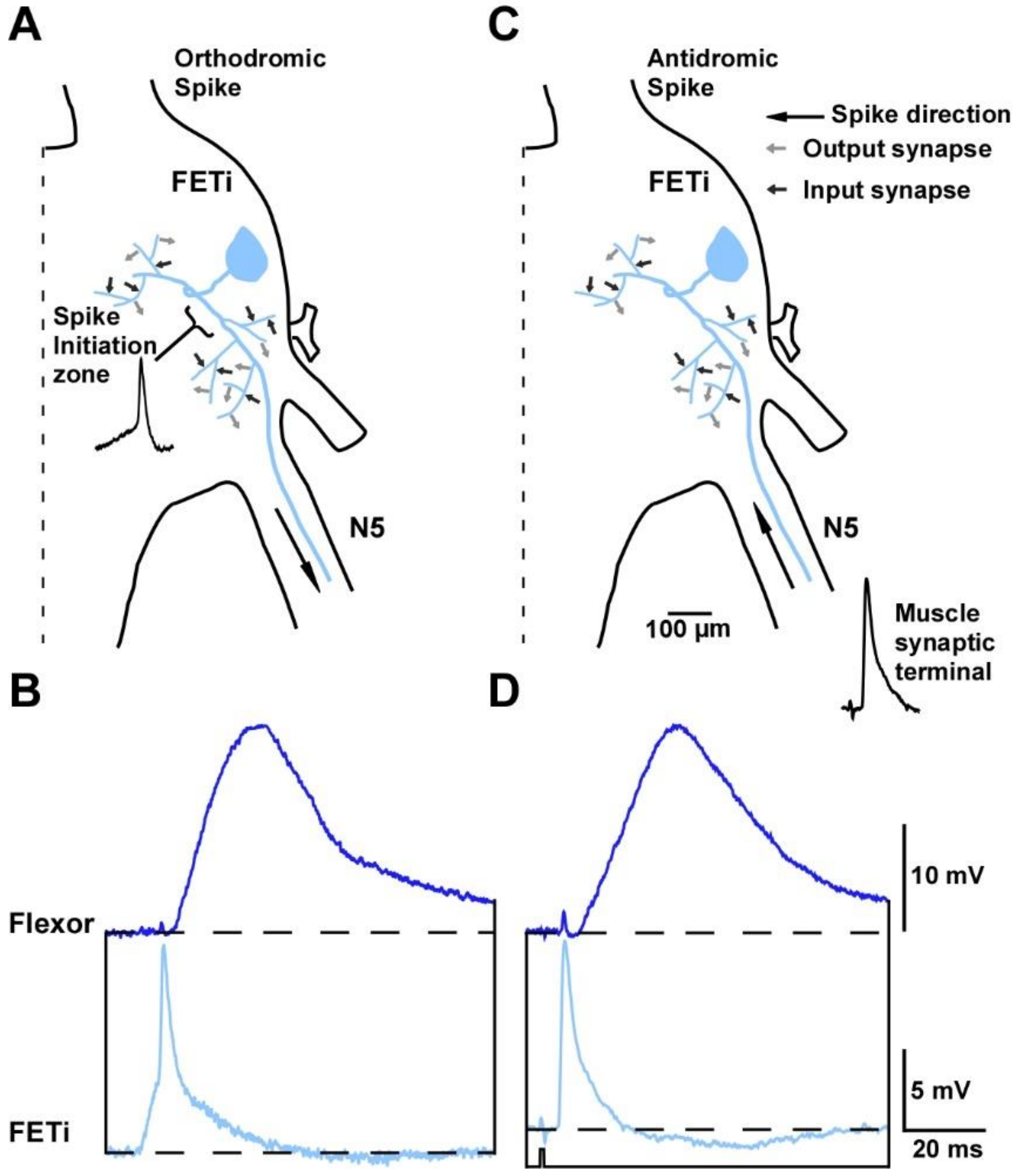

Supplemental Figure 1. Orthodromic and antidromic spikes in FETi evoke EPSPs in the flexors. (**A**) A diagram of FETi modified from a drawing of the branching morphology showing the central and peripheral output synapses, input synapses and spike initiation zone. Orthodromic spikes propagate from the primary neurite to the periphery and to the central arborizations evoking neurotransmitter release. This diagram is based on electrophysiological recordings[1,2] and transmission electron microscopy[3]. (**B**) A paired intracellular recording of FETi and a fast flexor. An orthodromic spike in FETi causes an EPSP in the flexor. (**C**) The same diagram as in A but showing the peripheral stimulus that evokes an antidromic spike. Antidromic spikes propagate from the peripheral terminals at the extensor tibiae muscle to the central arborizations evoking neurotransmitter release. This diagram is based on electrophysiological recordings[1,2] and transmission electron microscopy[3]. (**D**) A paired intracellular recording of FETi and a fast flexor. An antidromic spike in FETi causes an EPSP in the flexor.

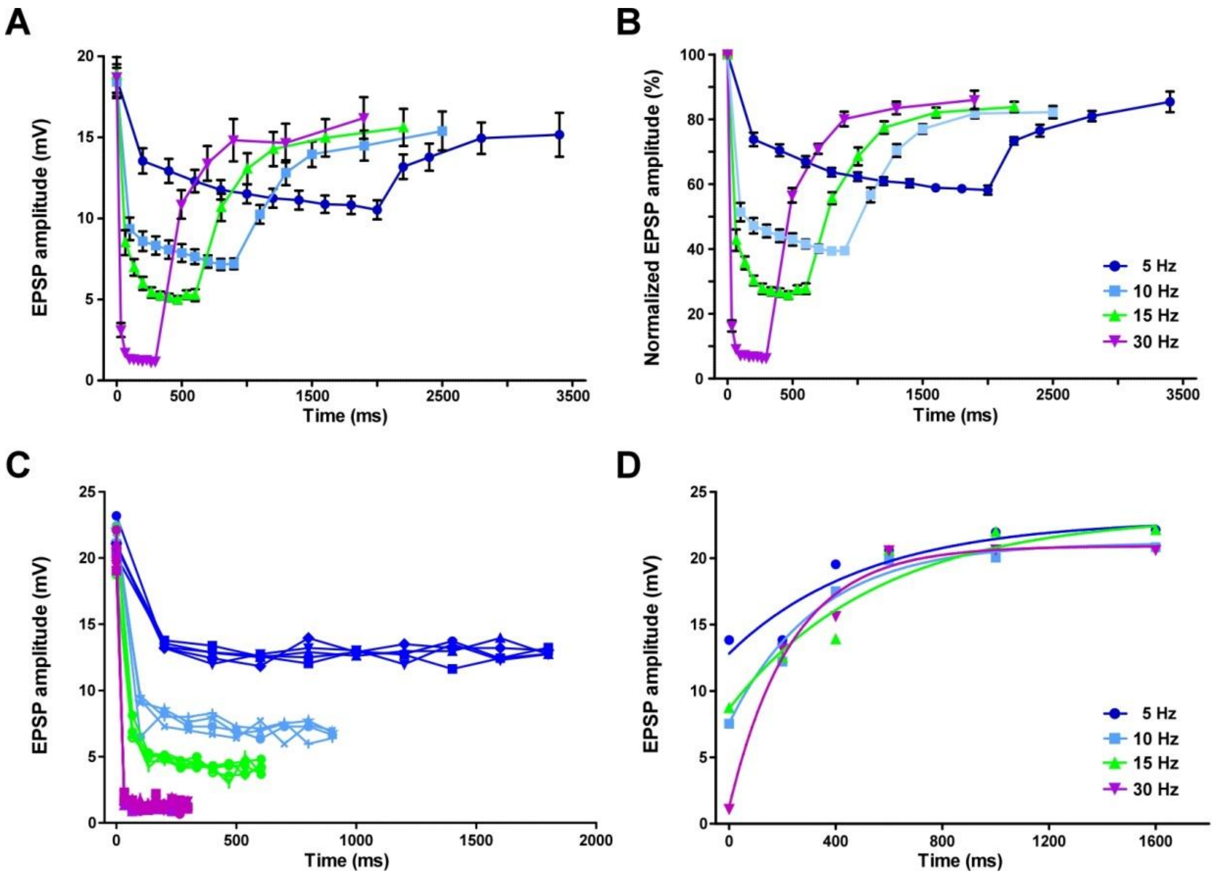

Supplemental Figure 2. Dynamics of short-term depression and recovery in the FETi-Flexor synapse. (**A**) The mean amplitudes of flexor EPSPs evoked by trains of 10 antidromic FETi spikes at between 5 and 30 Hz in standard saline. Error bars show the standard error of the mean (SEM). (**B**) The mean relative amplitudes of flexor EPSPs evoked by trains of 10 antidromic FETi spikes at between 5 and 30 Hz. Error bars show the standard error of the mean (SEM). (**C**) The amplitudes of EPSPs from a single flexor evoked by trains of 10 antidromic FETi spikes at between 5 and 30 Hz. Five repeats of each antidromic spike train are shown. (**D**) The amplitudes of EPSPs from a single flexor evoked by single FETi spikes to assess recovery from 200 to 1600ms. The recovery after trains of 10 antidromic FETi spikes at between 5 and 30 Hz is shown.

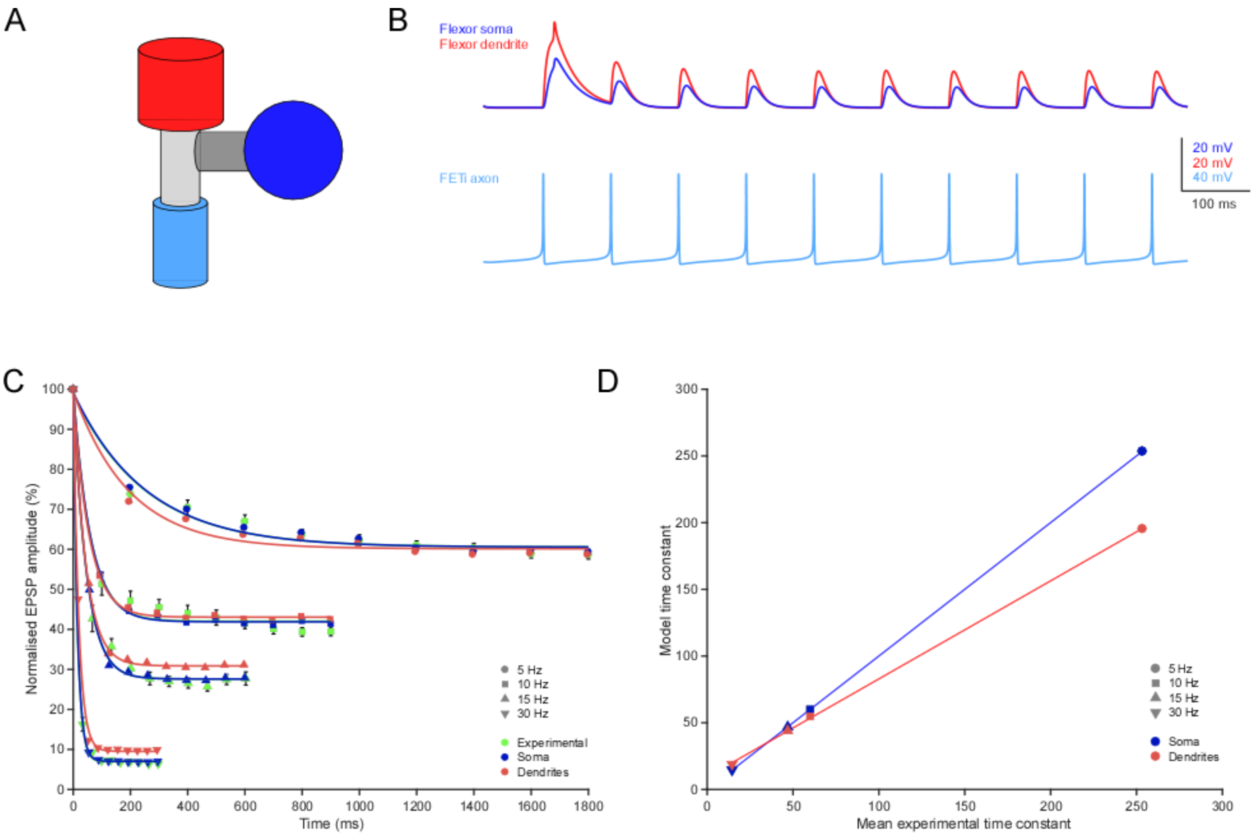

Supplemental figure 3. A multi-compartment model demonstrates that intracellular recordings from the flexor soma reflect synaptic dynamics in the dendritic branches. (**A**) A schematic diagram of the multicompartment flexor model. The dendritic compartment (red) is coupled to the primary neurite (pale grey), which is itself coupled to the axon (pale blue). A side branch from the primary neurite (dark grey) is coupled to the soma (dark blue). (**B**) Spikes in the presynaptic Fast Extensor Tibiae motor neuron (FETi) compartment elicits an excitatory post-synaptic potential in the flexor motor neuron dendrite, which spreads to the soma. The somatic EPSPs are attenuated with respect to those in the dendritic compartment. Sequential FETi spikes elicit smaller EPSPs in the flexor due to short-term depression. (**C**) The normalized amplitudes of EPSPs in the dendritic and somatic compartments of the flexor model undergo frequency dependent reduction that matches the reduction shown by flexor EPSPs recorded in vivo. Both in vivo and in silico EPSPs show frequency-dependent short-term depression that can be fitted by an exponential decay. (**D**) The time constants fitted to trains of flexor model EPSP amplitudes from the dendritic or somatic compartments are linearly related to those fitted to trains of flexor EPSP amplitudes recorded *in vivo*.

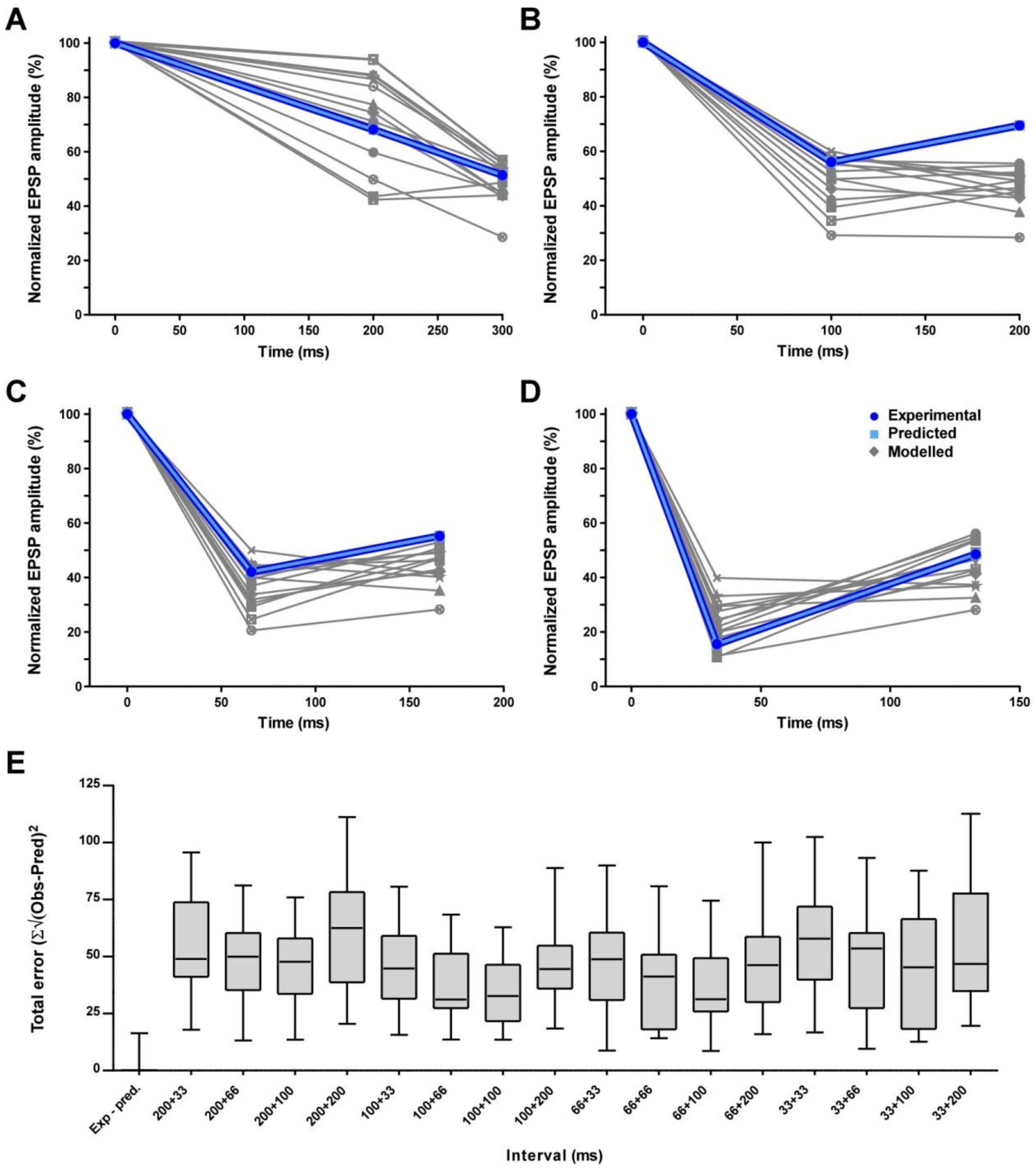

Supplemental Figure 4. The ability of the computational model to fit spike triplets. (**A**) The computational model fits (pale blue) to experimental flexor EPSP amplitudes (dark blue) evoked by a first interval of 200 ms and a second interval of 100 ms. Replacing the model parameters with those obtained from fits of other intervals prevents the model from fitting the experimental data. Model Parameter: U=1.02; A=97.71; τ_{rec} =125 ms. (**B**) As in A but with a first interval of 100 ms followed by a second interval of 100 ms. Model parameters: U=1.02; A=97.71; τ_{rec} =125 ms. (**C**) As in A but with a first interval of 66 ms followed by a second interval of 100 ms. Model parameters: U=0.98; A=101.04; τ_{rec}

=128 ms. (**D**) As in A but with a first interval of 33 ms followed by a second interval of 100 ms. Model parameters: U=1.03; A=96.35; τ_{rec} =145 ms. (**E**) A box chart of the error in the fit of the model to the experimental data compared with model predictions using parameters obtained from fits of other interval pairs. Even when the model is able to very accurately fit the experimental data, the fit is not 100% perfect. Therefore, the median of the Exp-pred values are near to zero. The error was calculated using 16 points for each interval. The error bar at the Exp-pred data point represents the maximum value 95% percentile.

Table S1. The frequency dependency of STD and recovery time constants

The time constants of STD and recovery are dependent upon the frequency of FETi spikes.

**Frequency** (Hz)	τ_{dep} (ms) (N=16)	τ_{rec} (ms) (N=13)
5	275.25±55.43	916.08±121.23
10	82.11±16.64	370.81±38.52
15	47.63±6.15	288.14±39.03
30	14.65±1.29	193.20±19.96

Table S2. The frequency dependency of STD and recovery during triplets of spikes separated by different intervals

The average relative EPSP amplitude of specific variable interval by fixed interval. The values were fitted with a single exponential to obtain the time constant.

**Variable interval** (ms)	**Flexor EPSP amplitude** (%)			
	**Fixed interval (ms)**			
	**200** (N=12)	**100** (N=12)	**66** (N=12)	**33** (N=8)
33	11.95±1.07	10.30±0.82	9.80±0.78	12.01±1.54
66	43.95±2.11	42.64±2.44	36.70±1.69	36.45±2.61
100	60.33±2.78	58.67±2.76	57.24±2.16	55.43±2.81
200	74.05±2.56	73.96±2.58	75.38±1.99	74.72±3.28
400	80.78±1.53	78.30±1.30	84.52±1.34	87.87±2.00
800	85.79±1.42	85.06±1.84	86.85±2.47	88.44±2.52
1600	86.59±2.07	83.70±1.29	85.13±2.25	86.13±2.09
**Time constant** (ms)	100.7±15.5	104.1±9.5	115.5 ±6.3	131.1±15.5

Table S3. Total error of the STD predicted fits for each stimulation frequency

The total error is obtained by comparing the experimental values of flexor EPSP amplitude versus the predicted EPSP amplitudes obtained by using the parameters of each frequency. Model parameters obtained by fitting the flexor EPSP amplitudes evoked by one frequency fail to predict accurately the EPSP amplitudes of the other frequencies.

Frequency (Hz)	Total error ($\sum\sqrt{(exp - model)^2}$) (mV)			
	Frequency (Hz)			
	5	10	15	30
5	17.94	53.28	65.08	63.49
10	135.17	30.79	60.84	112.24
15	69.72	73.26	14.68	41.80
30	66.50	93.37	42.32	28.34

References

1. Gwilliam, G. & Burrows, M. Electrical characteristics of the membrane of an identified insect motor neurone. *J. Exp. Biol.* **86,** 49–61 (1980).
2. Niven, J. E. & Burrows, M. Spike width reduction modifies the dynamics of short-term depression at a central synapse in the locust. *J. Neurosci.* **23,** 7461–9 (2003).
3. Burrows, M., Watson, A. H. D. & Brunn, D. E. Physiological and ultrastructural characterization of a central synaptic connection between identified motor neurons in the locust. *Eur. J. Neurosci.* **1,** 111–126 (1989).
